# Normalized Protein–Ligand Distance Likelihood Score for End-to-End Blind Docking and Virtual Screening

**DOI:** 10.1021/acs.jcim.4c01014

**Published:** 2025-01-17

**Authors:** Song Xia, Yaowen Gu, Yingkai Zhang

**Affiliations:** † Department of Chemistry, New York University, New York, New York 10003, United States; ‡ Simons Center for Computational Physical Chemistry at New York University, New York, New York 10003, United States; § NYU-ECNU Center for Computational Chemistry at NYU Shanghai, Shanghai 200062, China

## Abstract

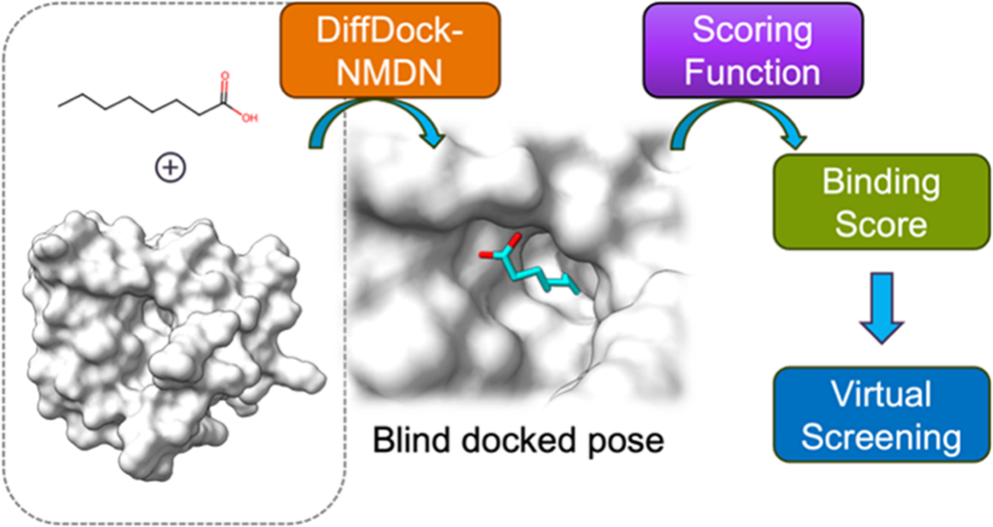

Molecular Docking is a critical task in structure-based virtual screening. Recent advancements have showcased the efficacy of diffusion-based generative models for blind docking tasks. However, these models do not inherently estimate protein–ligand binding strength thus cannot be directly applied to virtual screening tasks. Protein–ligand scoring functions serve as fast and approximate computational methods to evaluate the binding strength between the protein and ligand. In this work, we introduce normalized mixture density network (NMDN) score, a deep learning (DL)-based scoring function learning the probability density distribution of distances between protein residues and ligand atoms. The NMDN score addresses limitations observed in existing DL scoring functions and performs robustly in both pose selection and virtual screening tasks. Additionally, we incorporate an interaction module to predict the experimental binding affinity score to fully utilize the learned protein and ligand representations. Finally, we present an end-to-end blind docking and virtual screening protocol named DiffDock-NMDN. For each protein–ligand pair, we employ DiffDock to sample multiple poses, followed by utilizing the NMDN score to select the optimal binding pose, and estimating the binding affinity using scoring functions. Our protocol achieves an average enrichment factor of 4.96 on the LIT-PCBA data set, proving effective in real-world drug discovery scenarios where binder information is limited. This work not only presents a robust DL-based scoring function with superior pose selection and virtual screening capabilities but also offers a blind docking protocol and benchmarks to guide future scoring function development.

## 1 Introduction

Predicting the binding pose of a small molecule ligand to a protein, known as molecular docking, is a crucial task in structure-based virtual screening.^1^ Conventional molecular docking methods rely on scoring functions and optimization algorithms that search for the global maximum of the scoring functions. While they offer satisfactory docking performance on known pocket docking tasks, they are typically computational expensive on blind docking tasks because of the vast search space when the exact binding pocket of the protein is unknown. Recently, Corso et al. introduced a diffusion generative model named DiffDock^2^ which learns the ligand binding pose distribution without assuming any prior knowledge of the protein pockets. By iteratively refining ligand poses through updates of translations, rotations, and torsion angles of the ligand molecule, DiffDock can sample binding poses from random initial ligand conformations. On the blind docking benchmark PDBBind, DiffDock significantly outperforms previous state-of-the-art DL-based and conventional molecular docking models for pose generation. However, DiffDock alone cannot be directly applied for virtual screening because the model itself does not provide an estimation of the protein–ligand binding strength.

Estimating the binding affinity between proteins and ligands is a pivotal procedure within structure-based virtual screening, particularly in molecular docking and postdocking strategies. Protein–ligand scoring functions serve as fast and approximate computational methods to evaluate the binding strength between them. Classical scoring functions typically employ a linear combination of force field or interaction descriptors to assess the binding affinity. Machine learning (ML)-based scoring functions possess the capability to learn more complex functional forms using ML algorithms. With the burgeoning popularity of DL^3^ methods, the emergence of DL methods has introduced new strategies for developing scoring functions in drug discovery.^1^ Initial attempts utilize hand-crafted features, including protein–ligand interactions and ligand-dependent descriptors, as input, applying multilayer perceptron to learn the binding affinity.^4−6^ As convolutional neural networks become more prevalent in the image processing domain, three-dimensional (3D) variants of the network have been proposed, utilizing vectorized 3D grids covering the binding region as input to predict the binding affinity.^7−12^ More recently, graph neural networks (GNNs) have been gaining significant attention in the materials science and chemistry domain, as molecules and interactions can be naturally represented with graphs.^13^ Many GNN-based scoring functions have been proposed, operating on the 3D geometries of protein–ligand binding complexes to predict binding affinity.^14−18^

In 2021, DeepDock^19^ introduced a geometric DL approach aimed at learning the statistical potential based on the distance likelihood. They employed a mixture density network (MDN) to predict the probability density distribution of the distance between ligand atoms and the protein surface. This integrated distance likelihood between each protein–ligand pair achieves competitive performance in docking and screening tasks. Subsequent development, such as RTMScore^20^ and GenScore^21^ use graph transformers for feature extraction, further enhancing performance and surpassing state-of-the-art ML- and DL-based scoring functions. However, these models rely on well-defined protein pockets generated from known binder structures, limiting their applicability in real-world virtual screening scenarios where the target protein may lack known binders. Additionally, the selected pocket is biased toward the binder ligand, potentially overlooking crucial interactions between decoy ligands and the protein, particularly when decoy structures are distant from the binder ligand. For example, Xia et al. have shown that RTMScore’s performance is less optimal when protein pockets are defined based on docked ligand binders, compared to when they are defined based on crystal ligand binders.^22^

In this study, in order to further investigate how well scoring functions perform on virtual screening applications without predefined protein binding pockets, we have developed an end-to-end DiffDock-NMDN blind docking and virtual screening protocol: for each protein–ligand pair, we employ DiffDock^2^ to generate multiple binding ligand poses based on the provided protein structure, followed by utilizing the Normalized Mixture Density Network (NMDN) score developed in this work to select the most suitable pose, and finally estimating the binding affinity using scoring functions. The NMDN score is a DL-based scoring function that effectively addresses several limitations present in existing methods. By harnessing the capabilities of large language models, we efficiently encode residue-level embeddings from entire protein sequences with the ESM-2 model.^23^ Inspired by the statistical potential method,^24−28^ we propose a reference term to normalize the MDN score used in recent works,^19−21^ which improves the robustness of the score to variations in protein–ligand cutoffs.

Evaluation on PDBbind time-split data set for blind docking reveals that the NMDN score effectively selects the optimal binding pose from multiple sampled poses. Since the NMDN score is trained purely on geometric information, we introduce an interaction module to leverage the experimental binding affinity data fully. The interaction module uses the learned protein and ligand representation, combined with the protein–ligand distance and ligand features to predict the experimental binding affinity as a regression task, providing complementary performance to the NMDN score. Extensive testing of the protocol on the CASF-2016 and Merck FEP data sets demonstrates competitive performances for both previously developed scoring functions and the scoring functions developed in this work, underscoring the potential of the DiffDock-NMDN protocol as a promising approach for blind docking. In real-world scenarios, our protocol achieves an average enrichment factor of 4.96 when applied to the 15 targets on LIT-PCBA data set using a selection approach combining the NMDN score and the binding affinity score, demonstrating to be effective for virtual screening applications targeting novel targets where known binder information is lacking. Corresponding source code is available at https://github.com/SongXia-NYU/DiffDock-NMDN and data sets are available at https://zenodo.org/records/11106356.

## 2 Methods

### 2.1 Model Overview

**Figure 1:**
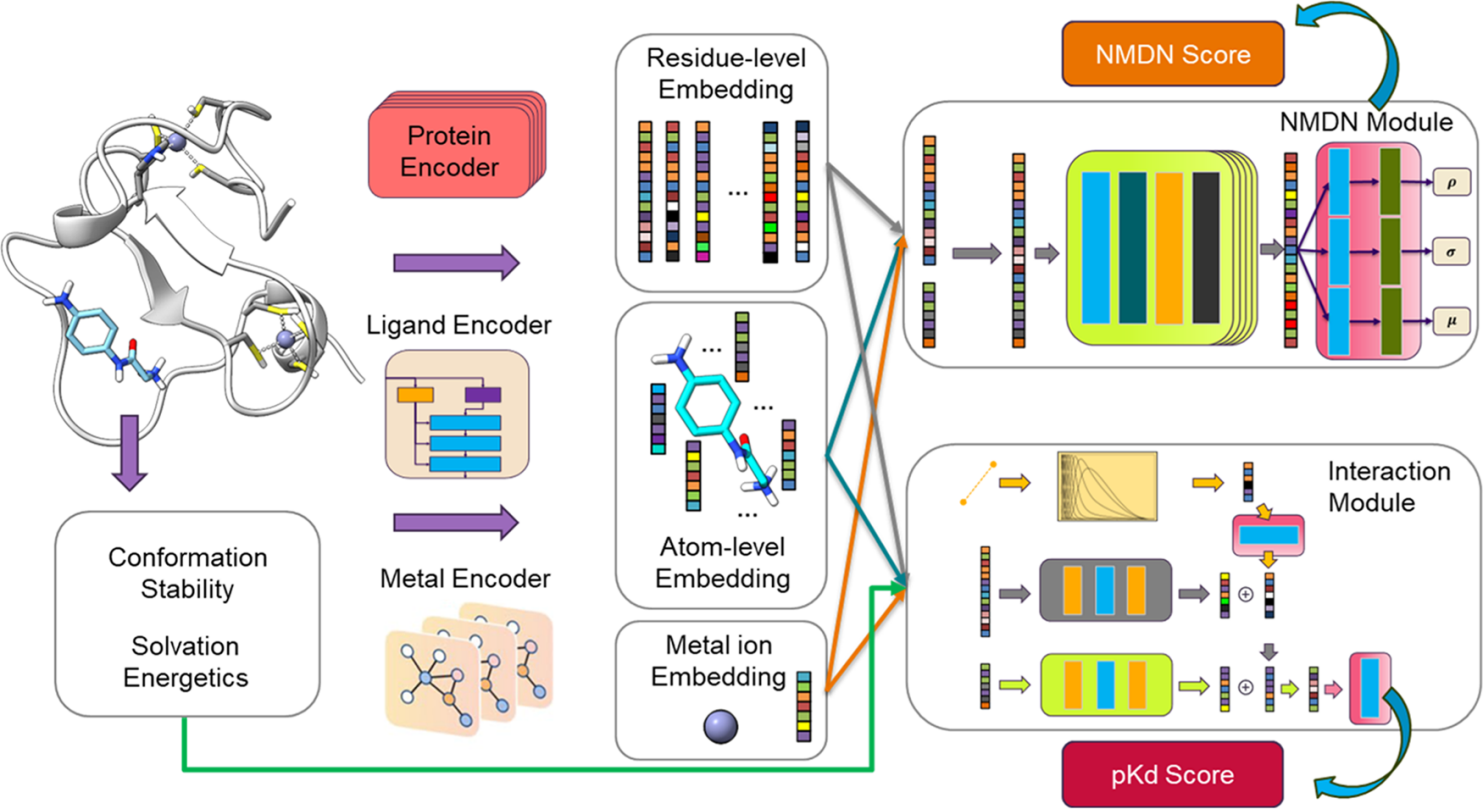
Overview of the scoring function proposed in this work. **Left**: The model uses the 3D geometry of the bound protein, metal, and ligand. Additionally, the ligand conformation stability and solvation energetics (Section 2.5) are computed and used by the interaction module. **Middle**: Three encoders are used to compute the embedding of protein residues, ligand atoms, and metal ions. **Right**: Two separate modules are used to compute the NMDN score and p*K*_d_ score separately.

Figure 1 illustrates the workflow of the model, wherein the protein–ligand binding pose serves as the input. The entire binding pose is considered a heterogeneous graph comprising protein residues at the residue level, ligand atoms, and metal ions at the atom level. We employ three separate encoders to encode the protein residues, ligand atoms, and metal ions (Section 2.2). Following encoding, we utilize an NMDN module to capture the protein–ligand and metal–ligand distance distribution (Section 2.3). To fully leverage the protein–ligand binding affinity information, we deploy an interaction module to predict the binding affinity as an auxiliary regression task (Section 2.4). Additionally, we generate supplementary ligand features such as conformation stability and solvation energetics to augment the model’s performance, serving as input for the interaction module (Section 2.5). For each binding pose, the model predicts an NMDN score, indicating its proximity to the crystal structure, and a p*K*_d_ score, estimating the experimental binding affinity.

### 2.2 Encoders for Protein, Ligand, and Metal

*Protein residue-level embeddings* are computed using the ESM-2 model.^23^ ESM-2 is a large language model trained on hundreds of millions of protein sequences. The learned protein representation has been demonstrated to be useful in a variety of protein-related tasks.^29^ For this study, we utilize the 650 million-parameter version of ESM-2. This model provides residue-level embedding, meaning each residue in the protein is embedded into a vector ***h***_***i***_^**prot**^. The ESM-2 model can encode every standard amino acid.
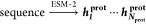
Where  is the *i*th protein residue embedding. *N*_prot_ is the number of residues in the protein. To mitigate computational costs, the ESM-2 embedding is precomputed and loaded from disk during training. Consequently, the ESM-2 model is not trained in this work.

*Ligand atom-level embeddings* are learned by the sPhysNet model.^30^ sPhysNet, a graph neural network developed from the PhysNet architecture,^31^ is part of the message passing neural network family.^32^ It takes 3D molecular geometries as input and generates atom-level embeddings. The generated embeddings have proven effective in predicting electronic energies,^33^ experimental hydration free energies, and experimental octanol–water partition coefficients.^30^ The model is pretrained in our previous study on the Frag20-solv-678k data set containing over 678 thousand molecular conformations with up to 20 heavy atoms. The model was trained on a multitask regression task with labels calculated at B3LYP/6-31G* level of theory with continuum solvent models. In this study, we initialize the model with pretrained weights and fine-tune it alongside other modules.

It takes the atomic numbers  and coordinates  of each atom and encodes them into vectors.
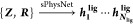
where *N*_lig_ is the number of atoms in the ligand.  is the *j*th ligand atom embedding. Given that the training set (as described in Section 4) includes atoms H, B, C, N, O, F, P, S, Cl, Br, and I, the model is able to encode these specific atom types.

*Metal ion embeddings* are computed by a model named knowledge graph-enhanced molecular contrastive learning with functional prompt (KANO).^34^ With element-oriented knowledge graph as a prior, the KANO model is pretrained through contrastive learning augmented by an element-guided graph. Pretraining is conducted on 250 thousand unlabeled molecules sampled from ZINC15.^35^ The KANO model has demonstrated exceptional performance across 14 downstream tasks.^34^ In this work, we integrated the KANO model to encode the metal ions in the system. Each metal ion is encoded into a vector . We initialize the KANO model with the published pretrained weights and fine-tune it alongside other modules.

### 2.3 Normalized Mixture Density Network Module

After encoding the protein, ligand, and metal ions, we utilize two normalized mixture density network (NMDN) modules to learn the distance distribution of protein–ligand and metal–ligand pairs.

#### 2.3.1 Protein–ligand NMDN Module Architecture

**Figure 2:**
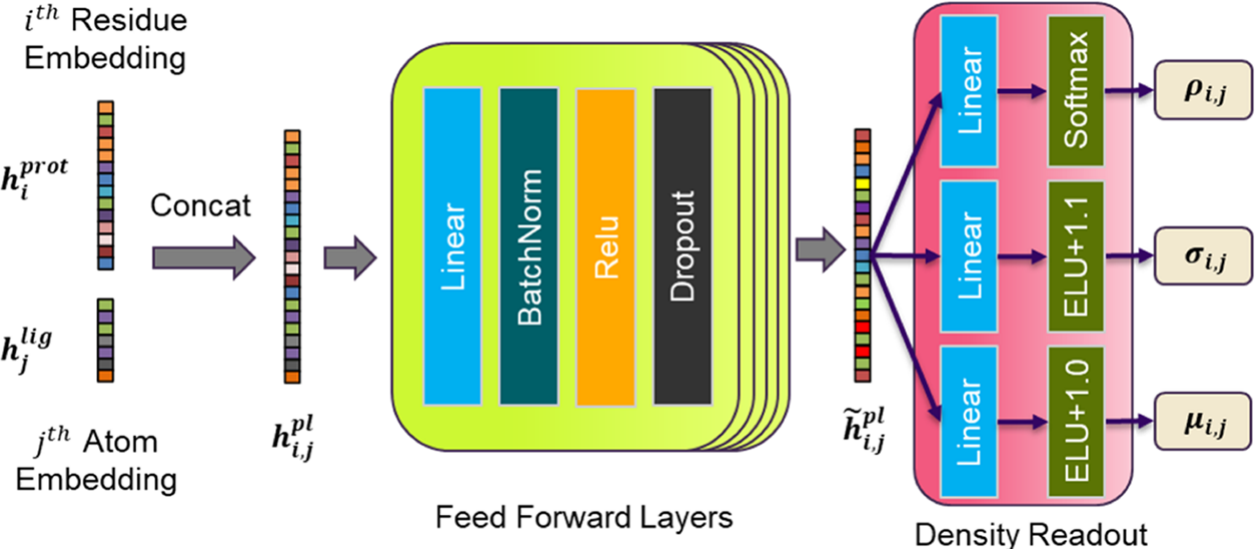
NMDN Module.

As shown in Figure 2, first the protein and ligand embeddings are concatenated.
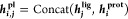
A multilayer perceptron (MLP) composed of 5 layers of linear transformation, batch normalization, RELU activation and dropout is used to further transform the concatenated embedding.
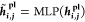


The hidden dimension of the MLP is set to 4096, as a result, . A density readout layer is then used to get the model prediction
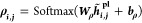

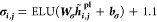

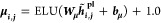
where  and  are trainable parameters in the linear transformation layers.

The predicted ,  and  parametrize the distribution of residue-atom minimum distance
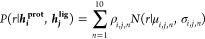
Where ρ_*i,j,n*_, μ_*i,j,n*_, and σ_*i,j,n*_ are the *n*th value of the predicted vector **ρ**_***i,j***_, **σ**_***i,j***_, and **μ**_***i,j***_, respectively. *N*(*r*|μ_*i,j,n*_,σ_*i,j,n*_) represents a normal distribution centered at μ_*i,j,n*_ with standard deviation σ_*i,j,n*_.

#### 2.3.2 Metal–Ligand NMDN Module

Similarly, a separate NMDN module is employed to predict the metal–ligand distance. This NMDN module has the identical architecture as the protein–ligand one except for minor differences in input dimensions and hidden representations.

#### 2.3.3 Training Loss for the NMDN Modules

For each protein–ligand pair, the loss function is defined as the negative logarithm of the predicted probability density at the actual distance between the protein and the ligand.
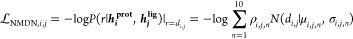
where *d*_*i,j*_ is the minimum distance between the *i*th protein residue and the *j*th ligand atom.

Similarly, for metal–ligand pairs, the loss function is defined as
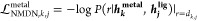
where *d*_*k,j*_ is the distance between the *k*th metal ion and the *j*th ligand atom.

All protein–ligand and metal–ligand pairs within a cutoff of 9 Å are trained. Therefore, the NMDN loss function is defined as
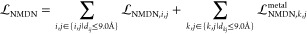


#### 2.3.4 Inference

For protein–ligand pairs, the NMDN score is defined as
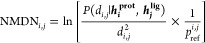


The reference probability *p*_ref_^*i,j*^ is computed by
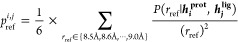
To compute the reference probability, instead of relying on a single reference point at the cutoff distance (9.0 Å), we sample six points near the cutoff distance (from 8.5 to 9.0 Å, at 0.1 Å intervals). This approach improves numerical stability, and the inclusion of the 1/6 factor ensures that the average probability is calculated over these six data points.

Similarly, the NMDN score for metal–ligand pairs is defined as
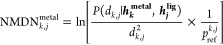
Finally, the NMDN score for the whole system is the summation of all protein–ligand and metal–ligand pairs within a cutoff of 9 Å.
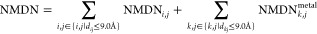
The NMDN score is used as the binding score for the system to evaluate ligand binding pose and perform virtual screening on the benchmark data sets.

### 2.4 Interaction Module

The interaction module estimates the experimental binding affinity from the protein–ligand binding pose. It serves two purposes. First, it provides an auxiliary task when training the NMDN score for more robust molecular representation learning. Second, it serves as a scoring function that is trained on experimental binding affinity data instead of geometric data in the NMDN score. We will demonstrate that combining these two scores helps us achieve better virtual screening ability than each score alone.

**Figure 3:**
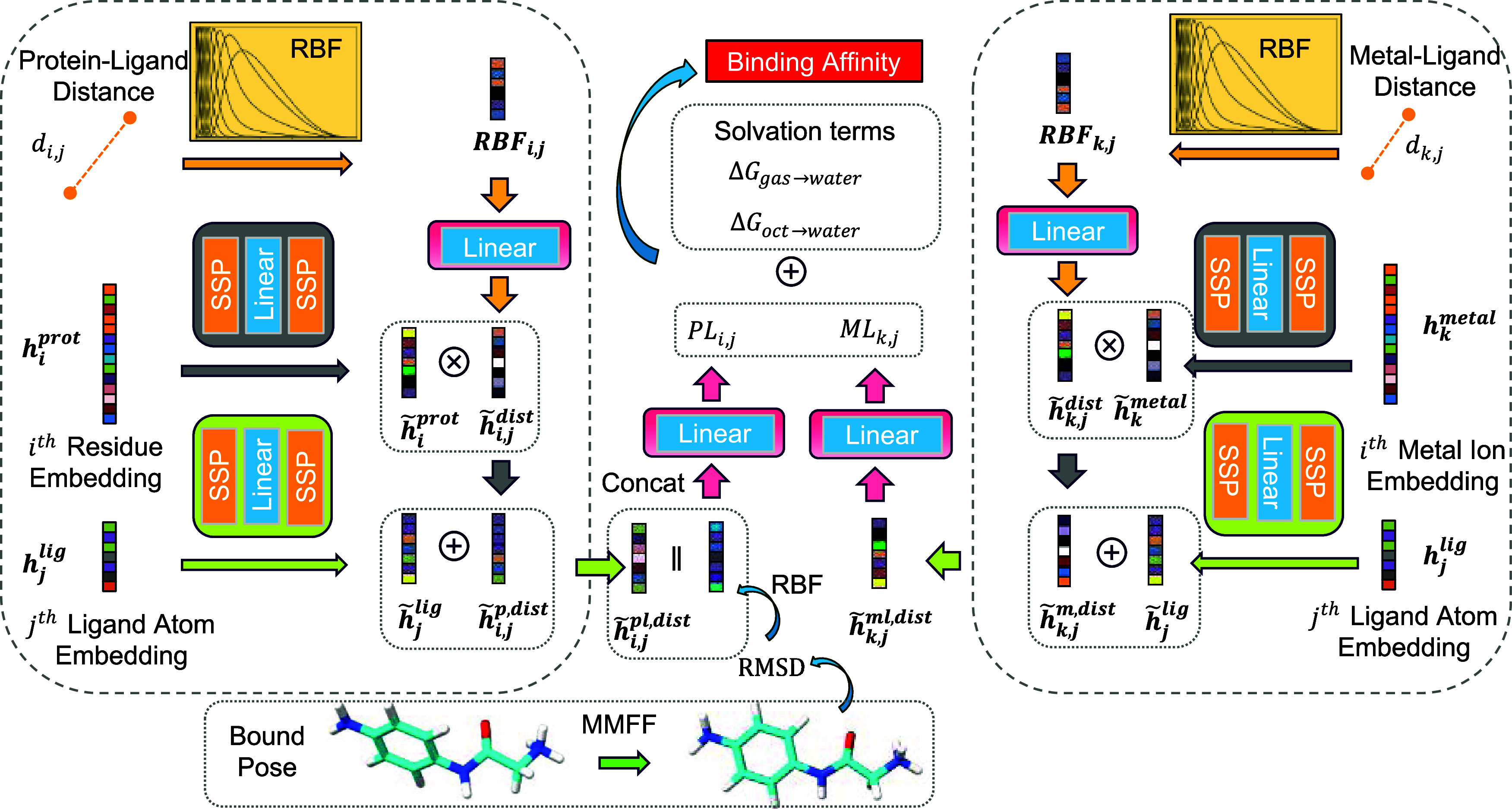
Interaction module for binding affinity prediction. **Left**: The calculation of the protein–ligand pair contribution; **Right**: the calculation of the metal–ligand pair contribution.

The module considers contribution from all protein–ligand and metal–ligand pairs within a distance of 9.0 Å. As illustrated in the left part of Figure 3, the protein–ligand pair contribution *PL*_*i,j*_ between the *i*th protein residue and the *j*th ligand atom is computed from the protein embedding ***h***_***i***_^**prot**^, ligand embedding ***h***_***j***_^**lig**^, the minimal distance between the protein residue and ligand atom *d*_*i,j*_, and the root-mean-squared distance (RMSD) described in Section 2.5. Similarly, the right part of Figure 3 depicts the computation of the metal–ligand pair contribution *ML*_*k,j*_. The binding affinity is then calculated based on the pairwise contributions and the solvation energetic terms described in Section 2.5. In the following paragraphs, we provide a detailed description of the components within the interaction module.

#### 2.4.1 Calculation of Protein–Ligand Pair Contributions

As illustrated in the left part of Figure 3, the protein–ligand  is first expanded into a vector by a series of Radial Basis Functions (RBFs).
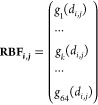

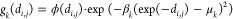
where *k* ∈ {1, 2, ···,64}. μ_*k*_ and β_*k*_ are learnable parameters that specify the center and width of *g*_*k*_(*d*_*i,j*_). The centers μ_*k*_ are initialized to K equally spaced values between e^–10^ and 1. The widths β_*k*_ are initialized to . ϕ(*d*_*i,j*_) is a smooth cutoff function defined as
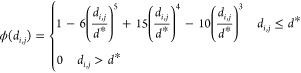
where d* = 10 Å.

The protein embedding  and the ligand embedding  are transformed by element-wise activation and linear layers.
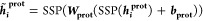

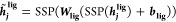
where ,  and . As a result, the transformed embeddings . SSP is the shifted softplus activation function which operates on every element of the vector.
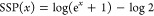


The distance information  is transformed by a linear layer.
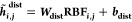
where  and .

The combined embedding ***h̃***_***i,j***_^**pl**,**dist**^ contains the protein residue, ligand atom, and the distance information
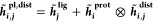
where ⊗ denotes the Hadamard product (element-wise product).

The predicted protein–ligand pair interaction PL_*i,j*_ is computed by a readout linear layer:
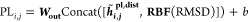
where  is the RMSD between the ligand crystal structure and the Merck molecular force field (MMFF)^36^ optimized ligand structure.  is the RBF expansion of the RMSD feature. Consequently, the concatenated dimension of ***h̃***_***i,j***_^**pl**,**dist**^**and RBF**(RMSD) is 192, and parameters  and  map the concatenated dimension into a scaler prediction .

*The calculation of metal–ligand pair contributions* is operated in a similar way. As illustrated in the right part of Figure 3, the calculation flow is nearly identical. Notably, the RMSD feature is not used for metal–ligand pair contributions.

*The binding affinity of the system (*p*K*_d_*-score)* is calculated based on the pairwise contributions and the solvation energetic terms (Section 2.5). Specifically, the predicted binding affinity is the summation of every protein–ligand and metal–ligand contribution, plus the solvation correction terms.
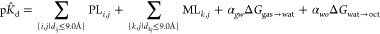


The coefficients of the solvation energetic terms are computed based on the hidden representation ***h̃***_**i**,**j**_^**pl**,**dist**^ by using two linear layers
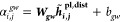

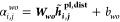
The average of the predicted pairwise α_*i,j*_^*gw*^, α_*i,j*_^*wo*^ over all protein–ligand pairs within 9.0 Å yields the coefficient α_*gw*_ and α_*wo*_, respectively.

The loss function for binding affinity is the mean-absolute-error (MAE) between the predicted p*K*_d_ value and the experimental p*K*_d_ value.
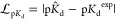


To train both NMDN module and interaction module simultaneously, we employ a training loss combining losses from both modules
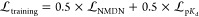


#### 2.4.2 Enhancing the Virtual Screening Capability of p*K*_d_ Score

Since the p*K*_d_-score is only trained on binders in PDBBind2020 data set (Section 4.1), it does not perform well when tasked to distinguish between binders and weak-binders. To overcome this limitation, we propose a method to enhance the virtual screening capability of the p*K*_d_ score. Our method involves preparing a data set of weak-binders (details in Section 4.1) and fine-tuning the interaction module of the p*K*_d_ score with a new loss function
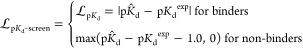
For binders, the loss function is the same as previously defined . For weak-binders, the module is tasked to predict a binding score lower than the experimentally measured binding affinity data. If the predicted score is lower than the experimental value, the loss is zero. Otherwise, the loss is the difference between the scores. To account for experimental measurement errors, we allow the module predicts up to p*K*_d_^exp^ + 1.0 without any penalty so the loss function for weak-binders is defined as max(p*K̂*_d_ – p*K*_d_^exp^ – 1.0, 0).

### 2.5 Ligand Conformation Stability and Solvation Energetics

Beside the protein–ligand binding pose, we incorporate additional ligand to enhance binding affinity prediction within the interaction module (Section 2.4).

Ligand conformation stability is assessed through the RMSD between the crystal bound ligand pose and force field optimized pose. For each protein-bound ligand, we optimize the pose using MMFF and subsequently calculate the RMSD between the optimized pose and the crystal pose.

The solvation energetics are represented by the transfer free energies of the ligand between gas, water, and 1-octanol. Specifically, for each ligand molecule, we compute the transfer free energy from gas to water Δ*G*_gas→water_ and the transfer free energy from 1-octanol to water Δ*G*_oct→water_ using our previously developed sPhysNet-MT model.^30^

## 3 DiffDock-NMDN Blind Docking and Virtual Screening Protocol

**Figure 4:**
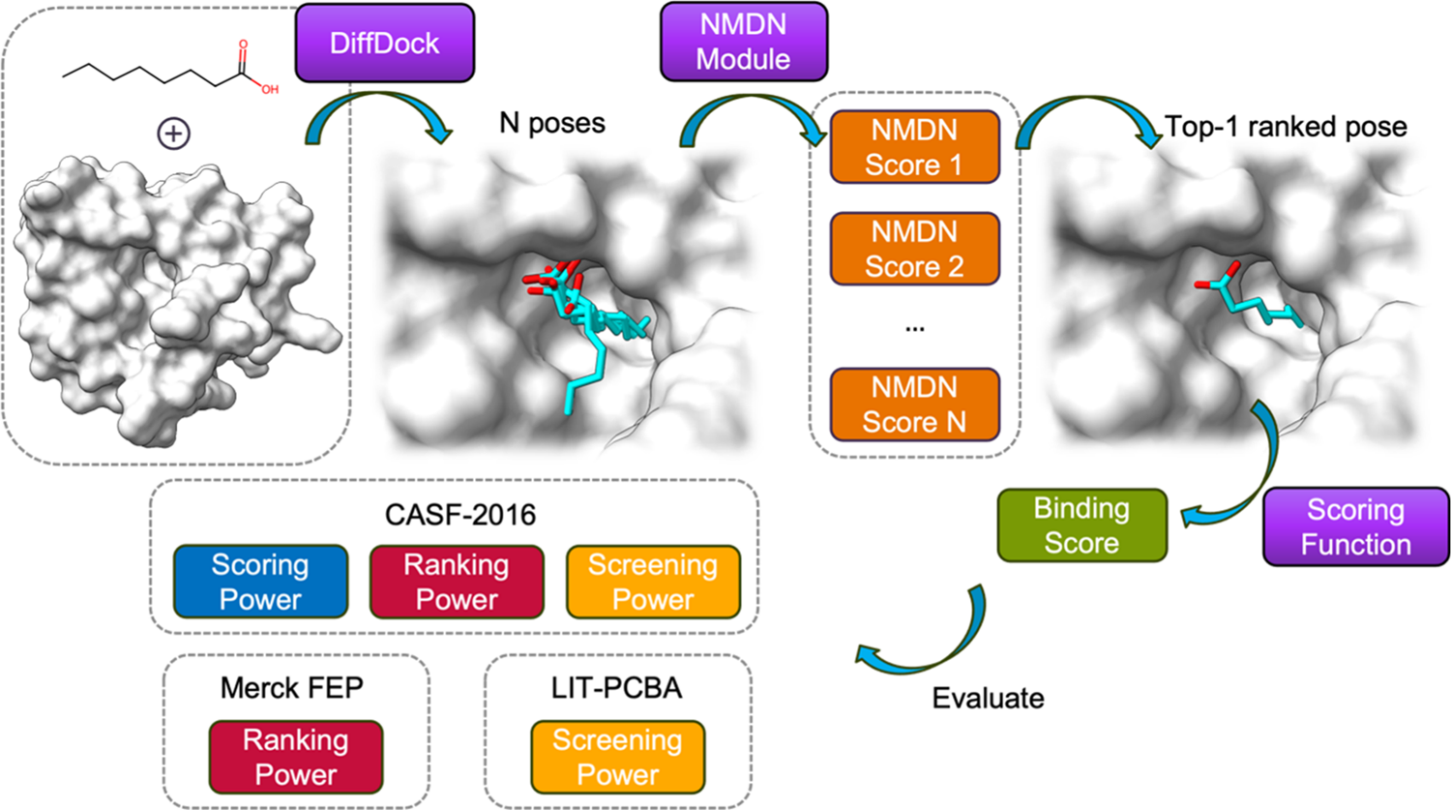
DiffDock-NMDN blind docking and virtual screening protocol.

Combining the NMDN score with the state-of-the-art diffusion model for ligand pose generation, we develop the DiffDock-NMDN blind docking and virtual screening protocol. Figure 4 shows the detailed workflow of this protocol. Starting from the two-dimensional (2D) molecular graph of the ligand and the 3D crystal structure of the protein, we employ DiffDock model to sample multiple poses. Those binding poses are evaluated by the NMDN module, and the top-1 ranked pose is selected as the docked pose for the specific protein–ligand pair. The selected pose can be further used by scoring functions to predict their binding score and evaluate the performances on several data sets. In this work, we not only evaluate the performances of the NMDN score from the NMDN module and the p*K*_d_ score from the interaction module, we also tested several representative scoring functions including AD4,^37^ Vina,^38^ Vinardo,^39^ Lin_F9,^40^ Δ_Lin_F9_XGB,^41^ RTMScore,^22^ and GenScore.^21^

## 4 Data Sets

### 4.1 Training Data Set Preparation

#### 4.1.1 Binder Data Set

The data set used in this study was constructed from the PDBBind database (version 2020),^42^ comprising 19,129 protein–ligand complexes. Additionally, we applied several filters as follows:

- Removal of covalent binders was performed using the script developed by Guo and Zhang^43^
- Structures with low resolution (cutoff of 2.5 Å) were excluded from the data set.
- Lin_F9 local optimization and scoring were conducted,^40^ and structures with excessively low Lin_F9 scores were filtered out. It is noteworthy that local optimization of ligand structures was solely utilized for Lin_F9 score computation, with the proposed model still utilizing crystal structures for training purposes.
- Ligand structures containing metal ions, which are less likely to represent drug molecules, were eliminated from consideration.
- PDB entries in the test sets were removed.

The data set was divided into training and evaluation subsets using the same train-evaluation split as described in the RTMScore^20^ work, resulting in 12,554 complexes for training and 1,083 complexes for evaluation. In the RTMScore study, the PDBBind data set was randomly split into training and validation sets. Due to the inherent randomness of this process, the RTMScore model was run with three independent splits. For simplicity, in this work, we utilized the first random split from the RTMScore study.

#### 4.1.2 Weak-Binder Data Set

The weak-binder data were collected from two crystal structure-free protein–ligand interaction bioactivity databases (EquiVS^44^ and Papyrus^45^) focusing on the activity types of K_i_ and IC_50_. The data in EquiVS and Papyrus were sourced and curated from multiple public databases, including ChEMBL,^46^ BindingDB,^47^ PubChem,^48^ Probe&Drugs,^49^ IUPHAR/BPS,^50^ EXCAPE,^51^ and literature data sets. To ensure high-quality weak-binder data, we implemented the following data processing and filtering strategies:

- Bioactivity data were converted to their negative logarithm, and only data with relation types “=” or “≤” (unit: neg.log of nM) were retained.
- Duplicate data present in both EquiVS and Papyrus databases were integrated, retaining only one instance. Conflicting data with variations greater than 1 neg. log. were excluded, while the remaining were relabeled using average values.
- Ligand structures were validated using MolVS^52^ and RDKit,^53^ with invalid data removed.
- Protein sequences were obtained from UniProt,^54^ and their full-length structures were predicted using AlphaFold2.
- Only data with bioactivity values lower than 5 neg. log. (equivalent to 10 μM) were retained and labeled as weak-binder data.

Following these criteria, a total of 250,267 protein–ligand pairs were identified as weak-binder data, comprising 137,417 unique molecules and 1,196 unique proteins, with no overlap with our adopted benchmarks. 60,000 protein–ligand pairs are randomly selected and the poses of them are generated by the DiffDock-NMDN blind docking protocol described in Section 3. The distribution of the K_i_ and IC_50_ is visualized in Figure S3.

### 4.2 Evaluation Metrics

#### 4.2.1 CASF-2016

The Comparative Assessment of Scoring Functions (CASF)^55^ benchmark is a widely recognized standard for assessing scoring functions. Specifically, we utilized the latest iteration of the benchmark, CASF-2016,^56^ which comprises 285 protein–ligand complexes across 57 distinct targets, each accompanied by crystal structures and experimental binding affinities. CASF-2016 evaluates scoring functions based on four key metrics: scoring power, ranking power, docking power, and screening power.

Scoring power is quantified by the Pearson’s correlation coefficient between predicted and experimental binding affinities. Ranking power is assessed through the average Spearman’s rank correlation coefficient across the 57 targets. Docking power is evaluated by the success rate of identifying a suitable binding pose within an RMSD threshold of 2 Å from a set of docked poses. Screening power is measured by the success rate and average enrichment factor in selecting true binders from the pool of 285 ligands. Crystal binding poses are employed to assess scoring and ranking power, whereas meticulously generated decoy ligand binding poses from molecular docking programs are utilized to evaluate docking and screening power.

Additionally, we implemented a DiffDock-NMDN blind docking protocol to assess the model’s performance without reliance on crystal or predocked poses. This protocol involves using DiffDock to generate 10 poses based on protein structures and ligand SMILES, followed by ranking these poses using the NMDN score. The top-ranked pose is then selected to evaluate docking power by computing the RMSD against the crystal pose. Furthermore, the p*K*_d_ score is computed using the top-ranked pose to evaluate scoring and ranking power.

*The Merck FEP benchmark*(57) is initially designed to assess the application of the FEP+ method^58^ in active drug discovery projects within an industry context. The data set consists of a total of 264 active ligands across eight pharmaceutically relevant targets and experimental binding affinity data curated from literature sources. The ligand poses are generated using either Flexible Ligand Alignment Tool or Glide core-constrained docking^59^ based on a reference ligand structure. The average Spearman’s rank correlation coefficient across the eight targets is used to measure the ranking power. The average is weighted by the number of ligands in the respective target. Similar to the blind docking evaluation conducted on the CASF-2016 benchmark, we also employ the DiffDock-NMDN blind docking protocol on the Merck FEP benchmark to measure the ranking performance without predocked poses.

*PDBbind time-split data set* serves as a test set to assess the performance of various molecular docking programs^2,60,61^ and protein–ligand scoring functions.^62^ This test set, derived from PDBBind2020 through a time-based split, comprises a subset of 1512 PDB entries recorded in or after the year 2019. From this subset, 363 entries were randomly sampled to form the final set, ensuring a manageable size for comparison with more computationally intensive docking programs. For each protein–ligand pair within PDBbind time-split data set, DiffDock employs a strategy where it samples multiple poses and subsequently utilizes a distinct confidence model to rank these poses. The top-ranked pose(s) are then selected to compute the RMSD to the crystal pose. The median RMSD and the success rate of generating a pose within an RMSD threshold of 2 Å are adopted as the primary metrics for evaluating docking performance. In this study, we introduce the DiffDock-NMDN protocol framework by replacing its existing confidence model with the NMDN score to evaluate the efficacy of the NMDN score in terms of its ability to select the most optimal binding pose from the sampled poses.

*LIT-PCBA* is an unbiased data set specifically designed for virtual screening and machine learning.^63^ It consists of 15 targets with over 10,030 true actives and 2,798,737 true inactives verified by experiments. The data set mimics the challenging real-world virtual screening scenarios in terms of hit rate and potency distribution. To generate binding poses between proteins and ligands, we employ the DiffDock-NMDN protocol. For each protein–ligand pair, DiffDock samples 10 poses, with the top-ranked pose determined by the NMDN score selected as the docked pose. It is worth mentioning that certain targets may have multiple PDB templates; however, for computational efficiency, we adhere to the approach outlined in the GenScore work^21^ by selecting only one target as the protein structure for docking purposes. Following pose generation, we evaluate the docked poses using either the NMDN score or p*K*_d_ score. The enrichment in true actives at a constant 1% false positive rate over random picking (EF_1%_) is used as the evaluation metric.

## 5 Results and Discussion

### 5.1 Benchmark Results

The CASF-2016 and Merck FEP benchmarks offer crystal or docked protein–ligand binding poses for evaluating scoring functions. Although this work primarily concentrates on blind docked poses, it is valuable to include benchmarking against these standard data sets to facilitate comparisons with other models.

#### 5.1.1 CASF-2016

Table S3 presents the performance comparison of the NMDN score against selected ML-based scoring functions. The NMDN score achieves a docking success rate of 0.856, a forward screening enrichment factor of 34.38, and a forward screening success rate of 66.7%, demonstrating competitive performance relative to state-of-the-art ML-based scoring functions. For a comprehensive list of scoring functions including both traditional and ML-based approaches and their docking and screening power, please refer to Figure S1.

Previous ML-based models that rely on statistical potentials, such as DeepDock, RTMScore, and GenScore, employ specific sets of cutoffs in their training and inference processes. Typically, all protein–ligand pairs within a cutoff distance of 7 Å are utilized during model training, whereas only pairs within 5 Å are considered during inference. To investigate the impact of different cutoff distances, we tested RTMScore across a range of cutoff values from 3 to8 Å. Table S1 demonstrates that RTMScore performs optimally when the test cutoff falls within the range of 4–6 Å, with a notable decline in performance observed outside this range. In contrast, for the NMDN score, we conducted experiments with various train/test cutoff combinations, including 10/8, 9/7, 8/6, 7/5, 10/10, 9/9, and 8/8 Å. As shown in Table S2, the NMDN score consistently exhibits robust performance across different sets of cutoff distances.

Since the binding affinity data is not directly utilized during the training of the NMDN score, we employ a separate interaction module to directly predict the binding affinity (p*K*_d_ score) through a regression task. Table S4 provides a performance comparison of the p*K*_d_ score against selected ML-based scoring functions. The p*K*_d_ score achieves a scoring power of 0.866 and a ranking power of 0.758, demonstrating competitive performance relative to state-of-the-art ML-based scoring functions. For a detailed overview of scoring functions, encompassing both traditional and ML-based approaches, and their respective scoring and ranking performance on the CASF-2016 benchmark, please refer to Figure S2.

#### 5.1.2 Merck FEP

As detailed in Section 4.2, the Merck FEP benchmark was initially crafted to evaluate the efficacy of the FEP+ method within active drug discovery endeavors, particularly within an industrial setting. Table S5 presents the performance of the p*K*_d_ score in comparison with other scoring functions and molecular modeling methods. The ranking power of the p*K*_d_ score varies across targets, with an average of 0.454 attained. For context, the FEP+ method, regarded as the gold standard in computing relative ligand-binding free energies, achieves an average ranking score of 0.65. It is notable that while most scoring functions achieve an average ranking power below 0.5, the p*K*_d_ score demonstrates competitive performance across the benchmark data set.

### 5.2 DiffDock-NMDN Blind Docking Protocol

In real-world virtual screening scenarios, the binding poses between proteins and ligands are typically unknown. Previous scoring functions have relied on traditional molecular docking programs to generate binding poses for evaluating virtual screening performance. In late 2022, Corso et al. introduced DiffDock, a diffusion generative model that learns the distribution of ligand poses from ligand and protein structures. On PDBbind time-split data set for blind docking, DiffDock achieves a top-1 success rate of 38%, significantly surpassing state-of-the-art molecular docking models. The DiffDock model comprises both a diffusion model and a confidence model. For each protein–ligand pair, the diffusion model generates 40 different poses, which are subsequently ranked by the confidence model to select the most promising pose. The confidence model is trained as a binary classification model, predicting whether the pose exhibits an RMSD to the crystal structure lower than 2 Å.

In the DiffDock-NMDN protocol, we replace the confidence model with the NMDN score and select the pose with the highest NMDN score. We apply the DiffDock-NMDN blind docking protocol to PDBbind time-split data set, CASF-2016, and MerckFEP benchmarks to assess the docking performance of the blind docking protocol. As illustrated in Table 1, the DiffDock-NMDN protocol achieves comparable performance to DiffDock in terms of top-1 success rate, and slightly better performance in terms of top-1 median RMSD.

**Table 1:** Blinding Docking Performance on PDBbind Time-Split Data Set, CASF-2016 Data Set, and Merck FEP Data Set^a^

	Top-1 RMSD(Å)		Top-5 RMSD(Å)		
method	%<2	Med.	%<2	Med.	reference
On PDBbind Time-Split Data Set					
Gnina^64^	22.9	7.7	32.9	4.5	(2)
Smina^65^	18.7	7.1	29.3	4.6	
Glide	21.8	9.3			
EquiBind	5.5	6.2			
TankBind	20.4	4.0	24.5	3.4	
P2rank^66^ + Smina	20.4	6.9	33.2	4.4	
P2rank + Gnina	28.8	5.5	38.3	3.4	
EquiBind + Smina	23.2	6.5	38.6	3.4	
EquiBind + Gnina	28.8	4.9	39.1	3.1	
DiffDock (10)	35.0 ± 1.4	3.56 ± 0.05	40.7 ± 1.6	2.65 ± 0.10	
DiffDock (40)	38.2 ± 1.0	3.30 ± 0.11	44.7 ± 1.7	2.40 ± 0.12	
DiffDock-NMDN (10)	34.6 ± 0.6	3.37 ± 0.11	41.1 ± 0.3	2.60 ± 0.05	this work
DiffDock-NMDN (40)	**38.4 ± 1.5**	**2.98 ± 0.07**	**45.4 ± 1.0**	**2.32 ± 0.10**	
On CASF-2016 Data Set					
DiffDock (10)	70.9	0.98	87.4	0.72	this work
DiffDock (100)	71.2	0.97	**89.8**	0.69	
DiffDock-NMDN (10)	76.1	0.99	88.8	0.71	
DiffDock-NMDN (100)	**76.8**	**0.90**	87.7	**0.67**	
On Merck FEP Data Set					
DiffDock (10)	**80.7**	1.15	88.4	0.85	This work
DiffDock-NMDN (10)	80.0	**0.99**	**89.1**	0.85	

a The integer number inside the parathesis represents the number of poses sampled for each protein-ligand pair. Best performance is marked in bold.

With the blind-docked poses generated from the DiffDock-NMDN protocol, we further evaluate the NMDN score and p*K*_d_ score in this work to evaluate the performance using blind docked poses. We also evaluate several representative scoring functions on the blind docked poses. Table 2 shows the performance of scoring functions on the CASF-2016 data set using the blind docked poses. The NMDN score achieves a forward screening power of 35.85 in terms of enrichment factor and 66.7% in terms of success rate. Meanwhile, the p*K*_d_ score achieves a scoring power of 0.799 and a ranking power of 0.646. Other selected scoring functions are also tested on our generated poses, showing competitive performance on various tasks. The low scoring-ranking power of the NMDN score is expected because the NMDN score is never trained on the binding affinity data. The low screening power of the p*K*_d_ score can be explained by the fact that p*K*_d_ score is only trained on binders, making it hard to distinguish between binders and weak-binders. When trained with weak binders added to the training set, p*K*_d_-screen does show an improvement in screening performance, with the enrichment factor increasing from 2.36 to 7.60. However, it should be noted that the NMDN score still achieves significantly higher screening performance than p*K*_d_-screen on the CASF-2016 data set.

**Table 2:** Performance of Representative Scoring Functions on the CASF-2016 Using the DiffDock-NMDN Blind Docked Poses^a^

		forward screening power			
pose	scoring	EF_1%_	success rate (%)	scoring power	ranking power
DiffDock-NMDN	NMDN	**35.85**	**66.7**	0.376	0.458
	p*K*_d_-score	2.36	7.0	**0.799**	**0.646**
	p*K*_d_-screen	7.60	28.1	0.731	0.579
	AD4^37^	11.36	35.1	0.178	0.302
	Vina^38^	15.18	42.1	0.340	0.323
	Vinardo^39^	19.34	54.4	0.314	0.346
	Lin_F9^40^	8.02	26.3	0.614	0.460
	Δ_Lin_F9_XGB^41^	11.27	40.4	0.730	0.479
	RTMScore^22^	27.93	56.1	0.444	0.440
	GenScore^21^	26.53	57.9	0.664	0.544

a Best performance is marked in bold.

Table 3 shows the performance of scoring functions on the Merck FEP data set using the blind docked poses. The p*K*_d_ score with blind-docked poses achieves an average ranking power of 0.39 across eight targets, while the NMDN score achieves an average ranking power of 0.26. Other selected scoring functions are also tested on the same set of poses, showing average ranking power ranging from 0.17 to 0.35. These results not only demonstrate the superior performance of the NMDN score as a scoring function, but also underscore the potential of the DiffDock-NMDN protocol as a promising approach for blind docking, generating reasonable docking poses for scoring functions to estimate the strengths of binding.

**Table 3:** Ranking Powers in Terms of Spearman Correlation Coefficient across Eight Targets on the Merck FEP Using DiffDock-NMDN Blind Docked Poses^a^

pose	scoring	hif2a	pfkfb3	eg5	cdk8	shp2	syk	cmet	tnks2	Avg
DiffDock-NMDN	NMDN	0.33	0.38	0.22	–0.15	0.65	–0.01	0.47	0.40	0.26
	p*K*_d_-score	0.32	0.38	0.37	0.13	0.44	0.46	0.40	0.67	**0.39**
	p*K*_d_-screen	0.38	0.45	0.00	0.31	0.23	–0.04	–0.01	0.22	0.20
	AD4^37^	0.32	0.29	–0.12	0.21	0.49	0.34	0.40	0	0.25
	Vina^38^	0.39	0.42	–0.45	–0.22	0.48	0.43	–0.01	–0.03	0.17
	Vinardo^39^	0.37	0.59	–0.26	–0.42	0.62	0.46	0.12	–0.11	0.20
	Lin_F9^40^	0.42	0.60	–0.18	0.36	0.93	0.51	0.04	0.31	0.35
	Δ_LinF9_XGB^41^	0.38	0.68	–0.18	0.33	0.61	0.35	0.04	0.43	0.33
	RTMScore^20^	0.35	0.61	0.19	–0.24	0.14	0.13	0.05	0.06	0.18
	GenScore^21^	0.14	0.46	0.17	0.03	0.28	0.30	0.16	0.08	0.21

a Best performance is marked in bold.

Finally, we assess our method on the LIT-PCBA data set to evaluate real-world screening performance. Table S6 presents statistics for the data set, highlighting a notable imbalance between the number of actives and inactives, mirroring real-world virtual screening scenarios. It is important to note that over half of the targets use cell-based phenotypic assays, so many actives are not validated against their presumed targets. Moreover, to reduce computational costs, we utilized only one PDB template per target, in consistent with the approach outlined in the GenScore article.

We employed the DiffDock-NMDN protocol to generate docked poses. For each protein–ligand pair, DiffDock sampled 10 poses, selecting the highest-ranked pose based on the NMDN score. The selected pose underwent further scoring using either the NMDN score or p*K*_d_ score, with the top 1% ranked ligands utilized to calculate the enrichment factor. Additionally, we combined both scores by selecting the top 0.5% ligands from each score and merging them. When there are overlaps in the top 0.5% of ligands from each score, the combined set of selected ligands is less than 1% of the total ligands. To address this, our selection algorithm incrementally adds additional ligands from each score, based on their ranking, one at a time. This process continues until the total number of unique selected ligands reaches 1% of the total ligand pool.

Table 4 displays the performance of these selection methods for each target, while Table 4 provides a comparative analysis of our model’s performance with other groups. The NMDN score achieves an average EF_1%_ of 3.90, while the p*K*_d_ score achieves an average EF_1%_ of 2.64. The combined selection method (p*K*_d_+NMDN) achieves an average EF_1%_ of 4.96. At EF_1%_ thresholds of >2, >5, and >10, the combined selection method satisfies 8, 3, and 3 targets, respectively. It is crucial to emphasize that our end-to-end blind docking protocol operates without leveraging known ligand binder information in either pose generation or score calculation. Consequently, the results in Table 4 cannot directly compare with previous screening performance listed in Table S7. Our method presents a suitable solution for virtual screening applications targeting novel targets where known binder information is lacking.

**Table 4:** Virtual Screening Performances on the LIT-PCBA Using DiffDock-NMDN Blind Docked Poses^a^

	individual score									combined score	
target	NMDN	p*K*_d_-score	p*K*_d_-screen	AD4	Vina	Vinardo	Lin_F9	Δ_LinF9_XGB	Genscore	p*K*_d_-score + NMDN	p*K*_d_-screen + NMDN^b^
ADRB2	11.77	0.00	5.88	0	0	5.88	0.00	0.00	5.88	11.77	11.76
ALDH1	1.35	1.67	1.41	1.63	1.52	1.35	1.70	1.66	1.33	1.52	1.45
ESR1-ago	0.00	7.69	15.39	0	7.69	7.69	0.00	7.69	0.00	0.00	15.38
ESR1-ant	1.96	2.94	0.00	0	1.96	4.9	3.92	1.96	2.94	0.98	1.96
FEN1	2.44	2.44	1.36	1.08	2.17	1.9	0.81	0.81	0.54	3.79	2.71
GBA	3.61	2.41	3.01	3.01	5.42	4.82	4.22	4.22	4.22	3.01	2.41
IDH1	0.00	12.82	0.00	5.13	5.13	7.69	12.82	10.26	0.00	12.82	0
KAT2A	3.09	1.03	2.58	1.03	0	1.03	0.52	0.00	0.00	3.09	4.12
MAPK1	2.27	0.65	0.97	1.62	0.65	2.6	0.97	0.97	3.90	1.62	1.3
MTORC1	0.00	0.00	3.09	0	1.03	1.03	0.00	1.03	1.03	0.00	1.03
OPRK1	0.00	0.00	0.00	4.17	0	8.33	0.00	0.00	0.00	0.00	0
PKM2	0.18	1.47	0.92	0.73	1.1	1.1	1.28	1.65	0.92	0.92	0.18
PPARG	25.93	0.00	3.70	11.11	7.41	0	3.70	3.70	11.11	25.93	25.93
TP53	3.80	1.27	0.00	0	0	0	0.00	1.27	0.00	3.80	3.8
VDR	2.04	0.45	1.36	0.34	0.79	1.47	0.45	0.45	0.90	1.58	2.38
average	3.90	2.32	2.64	1.99	2.32	3.32	2.03	2.38	2.18	4.72	**4.96**
#(EF_1%_ > 2)	**8**	5	6	4	5	7	4	4	5	7	**8**
#(EF_1%_ > 5)	2	2	2	2	**4**	**4**	1	2	2	3	3
#(EF_1%_ > 10)	2	1	1	1	0	0	1	1	1	**3**	**3**

a The performance is reported as the top 1% enrichment factor. Best performance is marked in bold

b We combined both scores by selecting the top 0.5% ligands from each score and merging them.

## 6 Ablation Study

### 6.1 Pose Selection Method

Since the DiffDock-NMDN protocol selects poses based on the NMDN score for each scoring function, there is a potential for bias when comparing different scoring functions. To address this, we benchmarked the models tested in this study using DiffDock-generated poses, allowing each scoring function to independently select the best pose. Table S8 summarizes the performance comparison on the CASF-2016 data set. While variations in model performance are observed—both increases and decreases—no significant trend emerges when comparing the two different pose sources for these scoring functions. Similarly, Table S9 summarizes the performance comparison on the MerckFEP. For the LIT-PCBA data set, however, we were unable to test all scoring functions on DiffDock poses due to the significantly higher computational cost—approximately 10 times that of DiffDock-NMDN poses. As a result, we have only tested three scoring functions on the LIT-PCBA data set. The corresponding results are presented in Tables S10. Moreover, we have made all poses generated by DiffDock for each protein–ligand pair available on Zenodo (see DATA AND SOFTWARE AVAILABILITY).

### 6.2 Components in the Interaction Module

Given the multiple components within the interaction module, we conducted an ablation study on the standard CASF-2016 data set. The results, summarized in Table S9, indicate that ligand features play an important role in enhancing the ranking power of the model (No.2 vs No.1). Since a significant portion of the protein structures in the training and test sets contain protein-bound metal ions, we included metal ions in our model to assess their contribution. However, it should be noted that adding metal ions does not significantly improve the model’s performance (No.10 vs No.2) on the CASF-2016 data set.

Additionally, we explored various protein encoding and metal encoding methods, including adding a linear layer or message passing layer after learning the protein/metal embeddings (No.3, No.7, No.8, No.11), using ESM2-GearNet^29^ for protein embedding (No.9), employing EquiformerV2^67^ for metal encoding (No.4), and utilizing the knowledge graph component of KANO for metal encoding (No.5).

### 6.3 Normalization Terms and Selection of Reference Distance

To investigate the effect of the normalization terms when computing the NMDN score, we evaluated the model’s performance using different reference distances on the CASF-2016 data set. Table S12 summarizes these results. When comparing scores without normalization terms (rows labeled as “None”), we observe an increase in the screening enrichment factor across all cutoffs when normalization is applied. Similarly, the screening success rate improves for all cutoffs, except for a single case where the training cutoff is 7.0 Å, the test cutoff is 5.0 Å, and the reference distance is 5.0 Å. However, no significant improvement in docking success rate is observed with the inclusion of the normalization term.

When analyzing the model’s performance across different reference distances with the same set of cutoffs, the NMDN score performs comparably. To simplify the model, we opted to set the reference distance equal to the test cutoff for further hyperparameter tuning, as shown in Table S2.

## 7 Conclusions

In this work, we propose a DL-based scoring function that generates NMDN scores based on statistical potentials and p*K*_d_ scores based on experimental binding affinities. The scoring function effectively addresses several limitations of state-of-the-art DL-based scoring functions, achieving superior performance on the CASF-2016 and MerckFEP benchmark. Leveraging the NMDN score within the DiffDock model, we develop a DiffDock-NMDN blind docking protocol that can efficiently sample protein–ligand binding poses without prior knowledge of protein pocket information. This blind docking protocol demonstrates effective on several benchmarks and achieves an average EF_1%_ of 4.96 on the LIT-PCBA data set.

Admittedly, the pose generation in the DiffDock-NMDN protocol is constrained by the capabilities of the DiffDock model. Recent advancements have shown that many DL-based docking methods, including DiffDock, may often produce physically implausible molecular structures.^68^ Postdocking energy minimization could further improve the poses generated by DiffDock, offering a promising way for enhancing docking performance.

Following the DiffDock model, numerous diffusion-based DL models for molecular docking have emerged. For instance, DiffBindFR enables flexible docking with ligand flexibility and protein pocket side chain changes. The DockGen model extends the DiffDock framework, leveraging a larger data set named BindingMOAD for training. Furthermore, NeuralPLexer integrates a diffusion process with essential biophysical constraints and a multiscale geometric DL system, enabling systematic prediction of binding ligand structures and their regulatory effects on protein folding. Our study highlights the promise of combining diffusion-based molecular docking models with the NMDN score for generating poses in real-world virtual screening applications.

## Data Availability

Corresponding source code is available at https://github.com/SongXia-NYU/DiffDock-NMDN and data sets are available at https://zenodo.org/records/11106356.

## Supporting Information Available

Docking and screening performance of the NMDN score on the standard CASF-2016 benchmark (Figure S1); scoring and ranking performance of the p*K*_d_ score on the standard CASF-2016 benchmark (Figure S2); bioactivity value distributions in the weak-binder data set (Figure S3); docking and screening performance of RTMScore on the standard CASF-2016 benchmark with different sets of cutoffs (Table S1); docking and screening performance of NMDN score on the standard CASF-2016 benchmark with different sets of cutoffs (Table S2); docking power and screening power of representative ML-based scoring functions on the standard CASF-2016 benchmark (Table S3); scoring power and ranking power of representative ML-based scoring functions on the standard CASF-2016 benchmark (Table S4); ranking powers in terms of Spearman correlation coefficient across eight targets on the standard Merck FEP benchmark (Table S5); data set statistics and selected PDB templates in LIT-PCBA (Table S6); LIT-PCBA screening performance of previous scoring functions (Table S7); model performance on the CASF-2016, letting the respective scoring functions choose the best pose, compared to using NMDN to select the pose (Table S8); model performance on MerkFEP, letting the respective scoring functions choose the best pose, compared to using NMDN to select the pose (Table S9); model performance on LIT-PCBA, letting the respective scoring functions choose the best pose, compared to using NMDN to select the pose (Table S10); ablation study over different components in the interaction module on the standard CASF-2016 data set (Table S11); ablation study over the reference distance selection on the standard CASF-2016 data set (Table S12) (PDF)

## Notes

The initial submitted version of this manuscript has been included as Chapter 4 of Song Xia’s Doctoral dissertation.^69^
