# Supplementary material for: Normalized Protein–Ligand Distance Likelihood Score for End-to-End Blind Docking and Virtual Screening
Source: J Chem Inf Model. 2025 Jan 17;65(3):1101–14. doi: 10.1021/acs.jcim.4c01014 (PMC11815853; doi:10.1021/acs.jcim.4c01014)
Supplement: Supplementary file 1 — ci4c01014_si_001.pdf [file ci4c01014_si_001.pdf]

Supporting Information

Normalized Protein-Ligand Distance Likelihood Score for End-to-end Blind Docking and Virtual Screening

Song Xia[1], *Yaowen Gu*[1], *and Yingkai Zhang*[1,2,3*]

[1]Department of Chemistry, New York University, New York, New York 10003, United States

[2]Simons Center for Computational Physical Chemistry at New York University, New York, New York 10003, United States

[3]NYU-ECNU Center for Computational Chemistry at NYU Shanghai, Shanghai 200062, China

Supplementary Figures

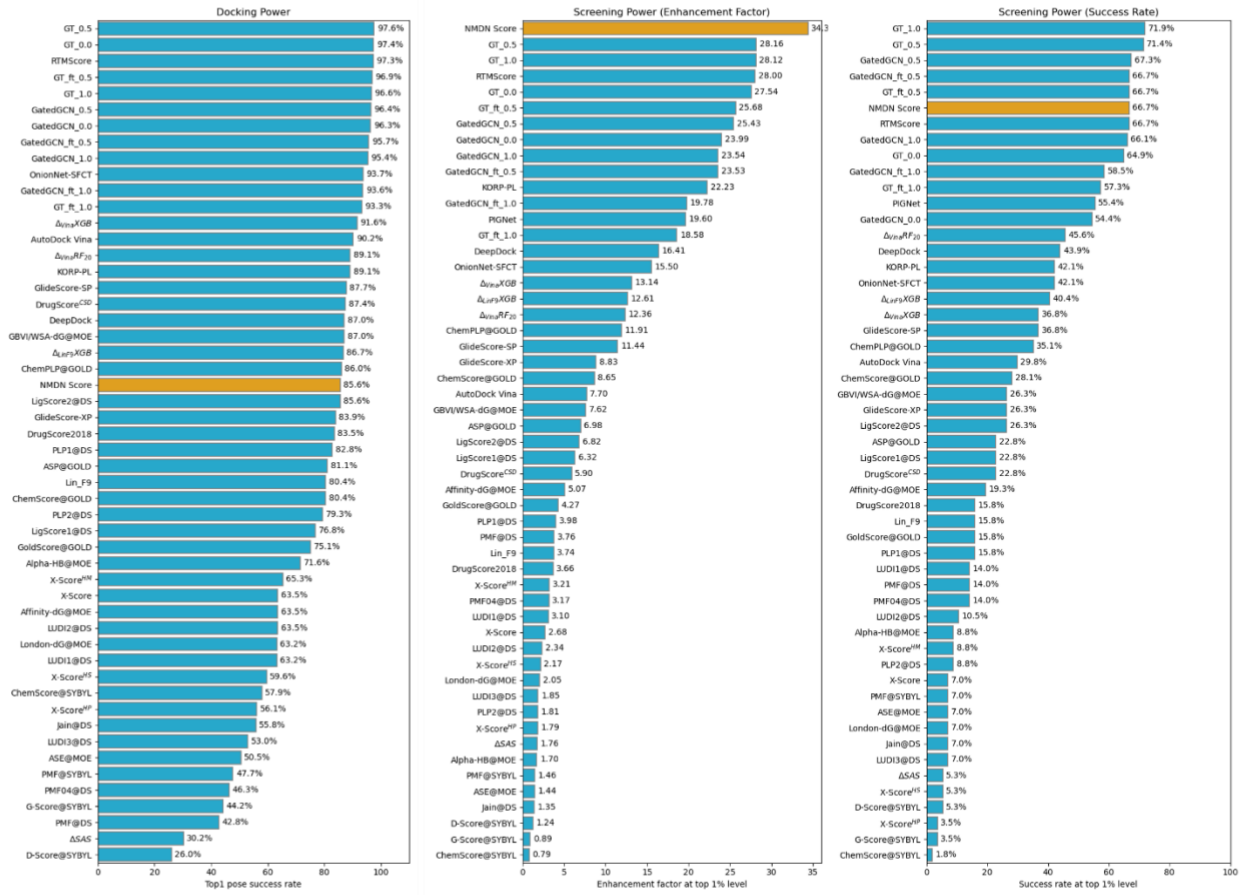

Figure S1. Docking and screening performance of the NMDN score on the standard CASF-2016 benchmark.

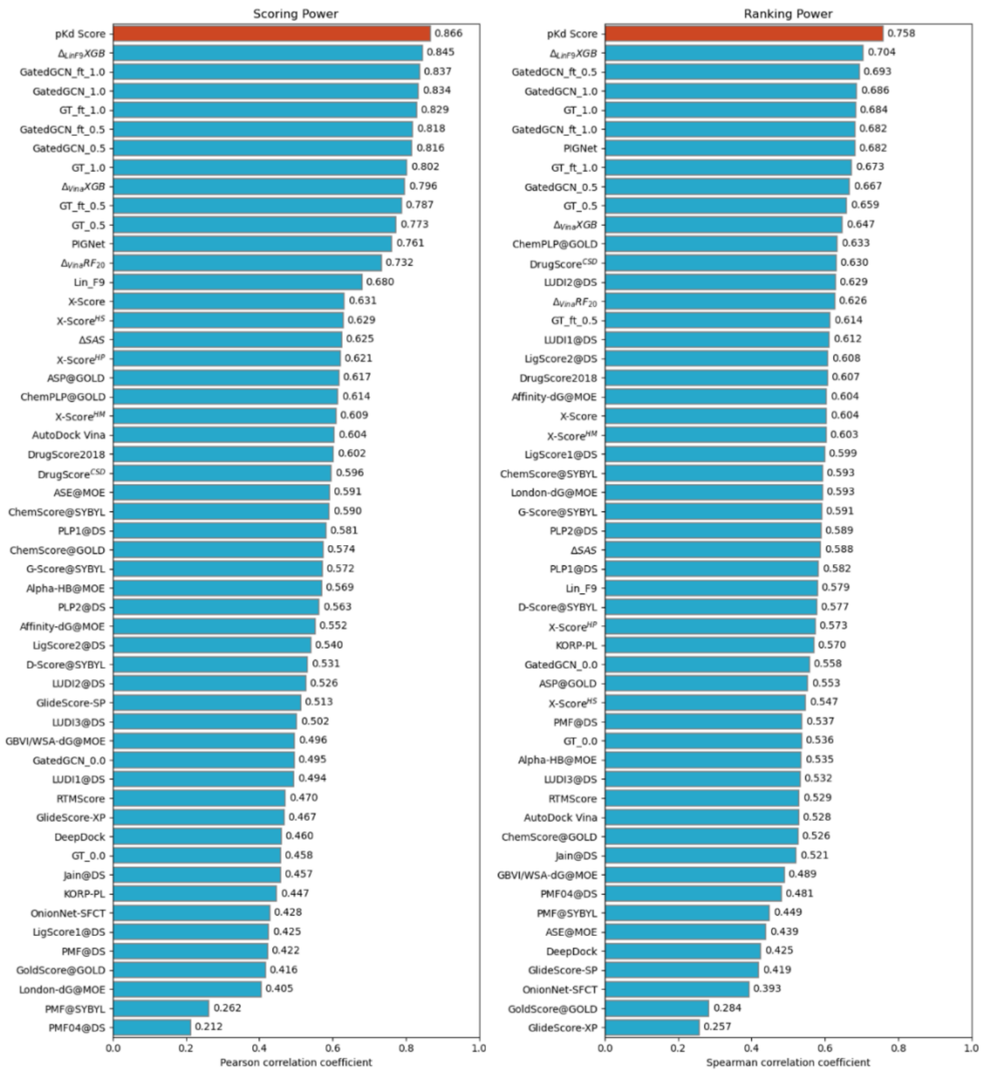

Figure S2. Scoring and ranking performance of the pKd score on the standard CASF-2016 benchmark.

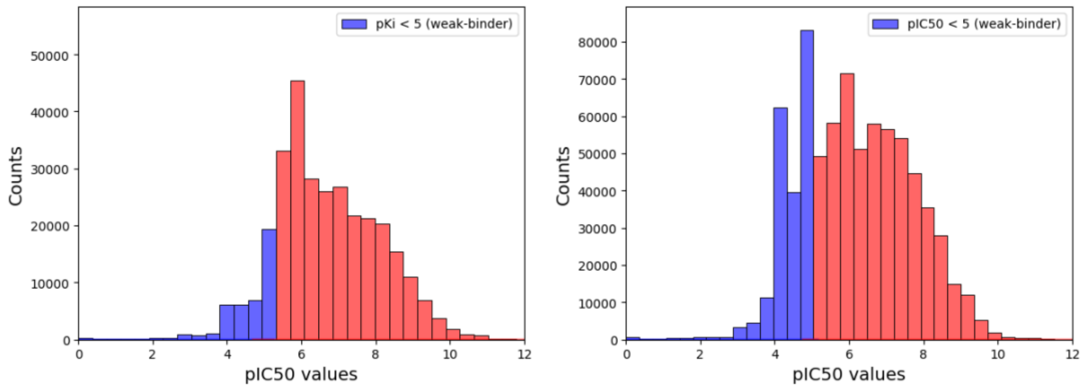

Figure S3. Bioactivity value distributions in the non-binder data set (non-binders are colored blue).

Supplementary Tables

Table S1. Docking and screening performance of RTMScore on the standard CASF-2016 benchmark with different sets of cutoffs.

MDN Cutoffs/Å		Docking Power w/o crystal pose*	Screen-EF1	Screen-SR1
Train	Test			
7.0	3.0	74.4%	14.970	29.8%
7.0	4.0	90.5%	27.640	63.2%
7.0	5.0	93.7%	28.78	73.7%
7.0	6.0	92.6%	25.010	59.6%
7.0	7.0	83.5%	10.790	33.3%
7.0	8.0	82.8%	10.640	35.1%

* The docking power here does not consider crystal poses, which is different from the performance reported in Table S3.

Table S2. Docking and screening performance of NMDN score on the standard CASF-2016 benchmark with different sets of cutoffs. The reference distance r_{ref} is set equal to the test cutoff, as discussed in section 6.3 of the manuscript.

NMDN Cutoffs/Å		Docking Power	Screen-EF1	Screen-SR1
Train	Test			
7.0	5.0	88.8%	31.23	61.4%
8.0	6.0	87.0%	33.96	75.4%
9.0	7.0	87.4%	35.02	66.7%
10.0	8.0	85.6%	34.17	64.9%
10.0	10.0	87.0%	31.77	63.2%
9.0	9.0	85.6%	34.38	66.7%
8.0	8.0	87.4%	32.61	66.7%

Table S3. Docking power and screening power of representative ML-based scoring functions on the standard CASF-2016 benchmark.

Model	Docking Power	Forward Screening Power		Reference
		$EF_{1\%}$	Success Rate	
NMDN Score	0.856	34.38	0.667	This work
RTMScore-unBiased	0.909	24.84	0.509	[1]
RTMScore	0.973	28.00	0.667	[2]
DeepDock	0.870	16.41	0.439	[3]
PIGNet	-	19.60	0.554	[4]
DeepBSP	0.885	-	-	[5]
OnionNet-SFCT	0.937	15.50	0.421	[6]
Δ_{Lin_F9}XGB	0.867	12.61	0.404	[7]
Δ_{Vina}XGB	0.916	13.14	0.368	[8]
$\Delta_{Vina}RF_{20}$	0.891	11.73	0.421	[9]
KORP-PL	0.891	22.23	0.421	[10]
GlideScore-SP	0.877	11.44	0.368	[11]
ChemPLP@GOLD	0.860	11.91	0.351	[12]
AutoDock Vina	0.902	7.70	0.298	[13]
GT_0.0	0.974	27.54	0.649	[14]
GT_ft_0.5	0.969	25.68	0.667	
GT_ft_1.0	0.933	18.58	0.573	
GT_0.5	0.976	28.16	0.714	
GT_1.0	0.966	28.12	0.719	
GatedGCN_0.0	0.963	23.99	0.544	
GatedGCN_ft_0.5	0.957	23.53	0.667	
GatedGCN_ft_1.0	0.936	19.78	0.585	
GatedGCN_0.5	0.964	25.43	0.673	
GatedGCN_1.0	0.954	23.54	0.661	

Table S4. Scoring power and ranking power of representative ML-based scoring functions on the standard CASF-2016 benchmark.

Model	Scoring Power	Ranking Power	Reference
pKd Score	0.866	0.758	This work
RTMScore	0.455	0.529	[2]
DeepDock	0.460	0.425	[3]
PIGNet	0.761	0.682	[4]
OnionNet-SFCT	0.428	0.393	[6]
$\Delta_{\mathrm{Lin_F9}}$XGB	0.845	0.704	[7]
Δ_{Vina}XGB	0.796	0.647	[8]
$\Delta_{\mathrm{Vina}}\mathrm{RF}_{20}$	0.739	0.635	[9]
KORP-PL	0.447	0.570	[10]
GlideScore-SP	0.513	0.419	[11]
ChemPLP@GOLD	0.614	0.633	[12]
AutoDock Vina	0.604	0.528	[13]
GT_0.0	0.458	0.536	[14]
GT_ft_0.5	0.787	0.614	
GT_ft_1.0	0.829	0.673	
GT_0.5	0.773	0.659	
GT_1.0	0.802	0.684	
GatedGCN_0.0	0.495	0.558	
GatedGCN_ft_0.5	0.818	0.693	
GatedGCN_ft_1.0	0.837	0.682	
GatedGCN_0.5	0.816	0.667	
GatedGCN_1.0	0.834	0.686	

Table S5. Ranking powers in terms of Spearman correlation coefficient across eight targets on the standard Merck FEP benchmark.

Method	hif2a	pfkfb3	eg5	cdk8	shp2	syk	cmet	tnks2	Avg	Reference
pKd Score	0.381	0.37	0.246	0.686	0.468	0.56	0.418	0.471	0.454	This work
AD4	0.376	0.53	−0.397	0.629	0.609	0.544	0.324	0.558	0.397	15
Vina	0.493	0.546	−0.520	0.849	0.569	0.519	−0.257	0.538	0.342	13
Vinardo	0.371	0.515	−0.475	0.782	0.490	0.379	−0.359	0.305	0.251	16
Δ_{LinF9}XGB	0.48	0.603	−0.099	0.826	0.640	0.103	0.077	0.458	0.386	7
X-Score	0.224	0.43	−0.316	0.406	−0.030	0.689	0.531	0.669	0.325	17
Pafnucy	0.224	0.43	−0.316	0.406	−0.030	0.689	0.531	0.669	0.325	18
GlideSP	0.445	0.48	−0.111	0.345	0.542	−0.006	0.378	0.316	0.299	11
GlideXP	0.41	0.513	0.017	0.617	0.490	0.124	0.165	0.582	0.365	19
FEP+	0.59	0.79	0.72	0.74	0.78	0.42	0.88	0.41	0.65	20
Prime-MM/GBSA	0.82	0.64	0.1	0.48	0.54	0.5	-0.12	0.22	0.38	
GT_0.0	0.317	0.544	0.116	0.665	0.537	0.074	0.693	0.512	0.432	14
GT_ft_0.5	0.357	0.45	0.210	0.671	0.608	0.230	0.693	0.54	0.47	
GT_ft_1.0	0.352	0.48	0.221	0.635	0.711	−0.006	0.617	0.555	0.446	
GT_0.5	0.459	0.59	0.204	0.682	0.445	0.099	0.772	0.58	0.479	
GT_1.0	0.437	0.571	0.275	0.675	0.338	0.144	0.677	0.578	0.462	
GatedGCN_0.0	0.398	0.533	0.132	0.685	0.575	0.106	0.610	0.464	0.438	
GatedGCN_ft_0.5	0.493	0.56	0.213	0.691	0.517	0.169	0.690	0.634	0.496	
GatedGCN_ft_1.0	0.519	0.578	0.206	0.712	0.609	0.214	0.727	0.586	0.519	
GatedGCN_0.5	0.395	0.58	0.221	0.679	0.490	0.121	0.746	0.61	0.48	
GatedGCN_1.0	0.455	0.635	0.293	0.693	0.489	−0.001	0.773	0.598	0.492	

Table S6. Dataset statistics and selected PDB templates in LIT-PCBA.

Target	#Actives	#Inactives	PDB Template
ADRB2*	17	312,483	4ldo
ALDH1	7,168	137,965	5l2m
ESR1-ago*	13	5,583	2p15
ESR1-ant*	102	4,948	2iok
FEN1	369	355,402	5fv7
GBA	166	296,052	2v3d
IDH1	39	362,049	4umx
KAT2A	194	348,548	5h86
MAPK1*	308	62,629	4zzn
MTORC1*	97	32,972	4dri
OPRK1*	24	269,816	6b73
PKM2	546	245,523	4jpg
PPARG*	27	5,211	5y2t
TP53*	79	4,168	3zme
VDR	884	355,388	3a2i

Table S7. LIT-PCBA screening performance of previous scoring functions. The poses are generated by traditional docking programs with known pocket information.

Group	Docking Programs	Scoring Function	Average $EF_{1\%}$	#Targets $EF_{1\%} > 2$	#Targets $EF_{1\%} > 5$	#Targets $EF_{1\%} > 10$
Sunseri et al.	Smina	RFScore-4	1.28	4	1	0
		RFScore-VS	0.73	5	2	0
		Vina	1.1	6	1	0
		Dense(affinity)	2.58	6	6	2
	Smina+Vinardo	Vinardo	0.99	4	2	0
Yang et al.	Smina + Lin_F9	Vina	2.78	6	2	1
		$\Delta_{Vina}RF_{20}$	3.18	6	3	2
		Lin_F9	2.21	8	1	0
		Δ_{Lin_F9} XGB	5.55	13	8	2
Tran-Nguyen et al.	Surflex	Surflex	2.51	6	3	0
		Pafnucy	5.32	9	7	3
		$\Delta_{Vina}RF_{20}$	5.38	10	7	3
		IFP	7.46	11	9	4
		GRIM	6.87	12	8	5
Shen et al.	Glide SP	GlideSP	4.06	9	4	1
		GT_0.0	6.51	10.33	5.33	3.00
		GT_ft_0.5	5.95	9.67	4.67	2.67
		GT_ft_1.0	6.41	9.67	6.00	3.33
		GT_0.5	5.69	11.00	5.00	2.00
		GT_1.0	5.24	9.33	4.67	2.33
		GatedGCN_0.0	6.22	8.67	5.00	3.00
		GatedGCN_ft_0.5	6.80	10.00	5.67	3.33
		GatedGCN_ft_1.0	6.27	8.67	6.33	3.00
		GatedGCN_0.5	5.53	9.00	5.33	2.33
		GatedGCN_1.0	5.14	8.33	5.00	2.00

Table S8. Model performance on the CASF-2016, letting the respective scoring functions choose the best pose, compared to using NMDN to select the pose. The best performance for each scoring function is marked in bold.

Scoring	Pose Generation	Pose Selection	Forward Screening Power		Scoring Power	Ranking Power
			$EF_{1\%}$	Success Rate		
Lin_F9[1]	DiffDock	NMDN	**8.02**	**26.3%**	0.614	0.460
		Lin_F9	6.81	22.8%	**0.651**	**0.516**
AD4[2]		NMDN	**11.36**	**35.1%**	0.178	0.302
		AD4	7.97	24.6%	**0.564**	**0.523**
Vinardo[3]		NMDN	**19.34**	**54.4%**	0.314	0.346
		Vinardo	15.59	45.6%	**0.512**	**0.439**
AutoDock Vina[4]		NMDN	**15.18**	**42.1%**	0.340	0.323
		AutoDock Vina	10.94	29.8%	**0.432**	**0.367**
Δ_{Lin_F9}XGB[5]		NMDN	11.27	40.4%	**0.730**	0.479
		Δ_{Lin_F9}XGB	**19.98**	**52.6%**	0.564	**0.486**
RTMScore[6]		NMDN	**27.93**	**56.1%**	0.444	**0.440**
		RTMScore	26.88	**56.1%**	**0.456**	0.437
GenScore[7]		NMDN	26.53	**57.9%**	**0.664**	**0.544**
		GenScore	**27.09**	50.9%	0.459	0.493

Table S9. Model performance on MerkFEP, letting the respective scoring functions choose the best pose, compared to using NMDN to select the pose.

Scoring	Pose Generation	Pose Selection	hif2a	pfkfb3	eg5	cdk8	shp2	syk	cmet	tnks2	Avg
Lin_F9[1]	DiffDock	NMDN	0.42	0.60	-0.18	0.36	0.93	0.51	0.04	0.31	0.35
		Lin_F9	0.49	0.58	-0.13	0.64	0.63	0.50	0.25	0.37	0.44
AD4[2]		NMDN	0.32	0.29	-0.12	0.21	0.49	0.34	0.40	0	0.25
		AD4	0.48	0.44	-0.02	0.52	0.41	0.26	0.70	0.03	0.36
Vinardo[3]		NMDN	0.37	0.59	-0.26	-0.42	0.62	0.46	0.12	-0.11	0.20
		Vinardo	0.47	0.63	-0.24	0.02	0.67	0.38	0.26	-0.14	0.29
AutoDock Vina[4]		NMDN	0.39	0.42	-0.45	-0.22	0.48	0.43	-0.01	-0.03	0.17
		AutoDock Vina	0.51	0.47	-0.47	0.38	0.62	0.40	0.11	-0.04	0.28
Δ_{Lin_F9}XGB[5]		NMDN	0.38	0.68	-0.18	0.33	0.61	0.35	0.04	0.43	0.33
		Δ_{Lin_F9}XGB	0.37	0.56	0.02	0.25	0.47	0.13	-0.11	0.17	0.25
RTMScore[6]		NMDN	0.35	0.61	0.19	-0.24	0.14	0.13	0.05	0.06	0.18
		RTMScore	0.32	0.45	0.14	0.03	0.22	0.36	0.26	0.27	0.27
GenScore[7]		NMDN	0.14	0.46	0.17	0.03	0.28	0.30	0.16	0.08	0.21
		GenScore	0.24	0.15	0.15	0.02	0.18	0.45	0.36	0.36	0.24

Table S10. Model performance on LIT-PCBA, letting the respective scoring functions choose the best pose, compared to using NMDN to select the pose.

Scoring	Vina		Vinardo		Lin_F9	
Pose Generation	DiffDock					
Pose Selection	NMDN	Vina	NMDN	Vinardo	NMDN	Lin_F9
ADRB2	0	0	5.88	0	0.00	0
ALDH1	1.52	1.19	1.35	1.3	1.70	1.37
ESR1-ago	7.69	7.69	7.69	7.69	0.00	0
ESR1-ant	1.96	3.92	4.9	4.9	3.92	3.92
FEN1	2.17	1.08	1.9	1.63	0.81	0.81
GBA	5.42	4.22	4.82	3.01	4.22	3.61
IDH1	5.13	2.56	7.69	0	12.82	10.26
KAT2A	0	1.03	1.03	1.03	0.52	1.55

MAPK1	0.65	0.65	2.6	2.92	0.97	1.62
MTORC1	1.03	0	1.03	1.03	0.00	0
OPRK1	0	0	8.33	0	0.00	0
PKM2	1.1	1.28	1.1	1.1	1.28	1.28
PPARG	7.41	7.41	0	7.41	3.70	3.7
TP53	0	0	0	1.27	0.00	0
VDR	0.79	1.02	1.47	1.24	0.45	0.34
Average	2.32	2.14	3.32	2.30	2.03	1.90
#($EF_{1\%} > 2$)	5	5	7	5	4	4
#($EF_{1\%} > 5$)	4	2	4	2	1	1
#($EF_{1\%} > 10$)	0	0	0	0	1	1

Table S11. Ablation study over different components in the interaction module on the standard CASF-2016 data set.

Number	Comment	Protein Encoding	Ligand Encoding	Metal Encoding	RMSD	Solvation	Score	Rank	Dock	Screen	
										EF1	SR1
1	-	ESM2	sPhysNet	✗	✗	✗	0.850	0.705	24.2%	2.14	5.3%
2	Add ligand features	ESM2	sPhysNet	✗	✓	✓	0.860	**0.768**	30.2%	--	--
3		ESM2+Linear	sPhysNet	✗	✓	✓	0.845	0.668	30.5%	--	--
4	Try different encoding methods	ESM2	sPhysNet	EquiformerV2	✗	✗	0.840	0.695	26.3%	1.89	3.5%
5		ESM2	sPhysNet	KG	✗	✗	0.832	0.677	27.0%	1.96	3.5%
6		ESM2	sPhysNet	KANO	✗	✗	0.851	0.711	25.6%	0.89	5.3%
7		ESM2	sPhysNet	KANO+MPNN	✗	✗	0.853	0.703	26.0%	0.54	1.8%
8		ESM2+MPNN	sPhysNet	KANO+MPNN	✗	✗	0.839	0.686	29.8%	0.89	1.8%
9		ESM2-GearNet	sPhysNet	KANO	✗	✗	0.355	0.421	62.8%	--	--
10	Combine best encoding with physical terms	ESM2	sPhysNet	KANO	✓	✓	**0.866**	0.758	27.0%	--	--
11		ESM2	sPhysNet	KANO+MPNN	✓	✓	0.827	0.687	35.8%	--	--

Table S12. Ablation study over the reference distance selection on the standard CASF-2016 data set. The reference probability is computed as the average of six reference distances to ensure numerical stability, consistent with the method described in Section 2.3 of the manuscript.

Train Cutoff	Test Cutoff	Reference	Docking Power	Screen-EF1	Screen-SR1
7.0 Å	5.0 Å	None	88.8%	29.12	64.9%
		6.5-7.0 Å	87.0%	32.25	70.2%
		6.0-6.5 Å	87.0%	34.46	70.2%
		5.5-6.0 Å	87.7%	35.9	71.9%
		5.0-5.5 Å	87.0%	33.27	66.7%
		4.5-5.0 Å	88.8%	31.23	61.4%
8.0 Å	6.0 Å	None	87.0%	30.00	66.7%
		7.5-8.0 Å	87.7%	32.94	68.4%
		7.0-7.5 Å	86.0%	33.78	70.2%
		6.5-7.0 Å	86.7%	34.71	75.4%
		6.0-6.5 Å	88.1%	33.08	73.7%
		5.5-6.0 Å	87.0%	33.96	75.4%
9.0 Å	7.0 Å	None	87.0%	30.02	61.4%
		8.5-9.0 Å	85.3%	32.33	64.9%
		8.0-8.5 Å	85.3%	33.72	68.4%
		7.5-8.0 Å	86.0%	33.72	66.7%

		7.0-7.5 Å	86.7%	33.53	63.2%
		6.5-7.0 Å	87.4%	35.02	66.7%
10.0 Å	8.0 Å	None	87.7%	31.72	63.2%
		9.5-10.0 Å	87.4%	33.76	66.7%
		9.0-9.5 Å	86.3%	34.44	63.2%
		8.5-9.0 Å	85.6%	35.38	63.2%
		8.0-8.5 Å	86.0%	35.44	64.9%
		7.5-8.0 Å	85.6%	34.17	64.9%

(1) Xia, S.; Chen, E.; Zhang, Y. Integrated Molecular Modeling and Machine Learning for Drug Design. *J. Chem. Theory Comput.* **2023**, *19* (21), 7478-7495. DOI: 10.1021/acs.jctc.3c00814.

(2) Shen, C.; Zhang, X.; Deng, Y.; Gao, J.; Wang, D.; Xu, L.; Pan, P.; Hou, T.; Kang, Y. Boosting protein–ligand binding pose prediction and virtual screening based on residue–atom distance likelihood potential and graph transformer. *Journal of Medicinal Chemistry* **2022**, *65* (15), 10691-10706.

(3) Méndez-Lucio, O.; Ahmad, M.; del Rio-Chanona, E. A.; Wegner, J. K. A geometric deep learning approach to predict binding conformations of bioactive molecules. *Nature Machine Intelligence* **2021**, *3* (12), 1033-1039.

(4) Moon, S.; Zhung, W.; Yang, S.; Lim, J.; Kim, W. Y. PIGNet: a physics-informed deep learning model toward generalized drug–target interaction predictions. *Chemical Science* **2022**, *13* (13), 3661-3673.

(5) Bao, J.; He, X.; Zhang, J. Z. H. DeepBSP—a Machine Learning Method for Accurate Prediction of Protein–Ligand Docking Structures. *J Chem Inf Model* **2021**, *61* (5), 2231-2240. DOI: 10.1021/acs.jcim.1c00334.

(6) Zheng, L.; Meng, J.; Jiang, K.; Lan, H.; Wang, Z.; Lin, M.; Li, W.; Guo, H.; Wei, Y.; Mu, Y. Improving protein–ligand docking and screening accuracies by incorporating a scoring function correction term. *Briefings in Bioinformatics* **2022**, *23* (3). DOI: 10.1093/bib/bbac051 (acccessed 3/12/2024).

(7) Yang, C.; Zhang, Y. Delta Machine Learning to Improve Scoring-Ranking-Screening Performances of Protein–Ligand Scoring Functions. *J Chem Inf Model* **2022**, *62* (11), 2696-2712. DOI: 10.1021/acs.jcim.2c00485.

(8) Lu, J.; Hou, X.; Wang, C.; Zhang, Y. Incorporating Explicit Water Molecules and Ligand Conformation Stability in Machine-Learning Scoring Functions. *J Chem Inf Model* **2019**, *59* (11), 4540-4549. DOI: 10.1021/acs.jcim.9b00645.

(9) Wang, C.; Zhang, Y. Improving scoring‐docking‐screening powers of protein–ligand scoring functions using random forest. *Journal of computational chemistry* **2017**, *38* (3), 169-177.

(10) Kadukova, M.; Machado, K. d. S.; Chacón, P.; Grudinin, S. KORP-PL: a coarse-grained knowledge-based scoring function for protein–ligand interactions. *Bioinformatics* **2020**, *37* (7), 943-950. DOI: 10.1093/bioinformatics/btaa748 (acccessed 3/12/2024).

(11) Friesner, R. A.; Banks, J. L.; Murphy, R. B.; Halgren, T. A.; Klicic, J. J.; Mainz, D. T.; Repasky, M. P.; Knoll, E. H.; Shelley, M.; Perry, J. K.; et al. Glide: A New Approach for Rapid,

Accurate Docking and Scoring. 1. Method and Assessment of Docking Accuracy. *Journal of Medicinal Chemistry* **2004**, *47* (7), 1739-1749. DOI: 10.1021/jm0306430.
(12) Korb, O.; Stützle, T.; Exner, T. E. Empirical Scoring Functions for Advanced Protein−Ligand Docking with PLANTS. *J Chem Inf Model* **2009**, *49* (1), 84-96. DOI: 10.1021/ci800298z.
(13) Trott, O.; Olson, A. J. AutoDock Vina: improving the speed and accuracy of docking with a new scoring function, efficient optimization, and multithreading. *Journal of computational chemistry* **2010**, *31* (2), 455-461.
(14) Shen, C.; Zhang, X.; Hsieh, C.-Y.; Deng, Y.; Wang, D.; Xu, L.; Wu, J.; Li, D.; Kang, Y.; Hou, T. A generalized protein–ligand scoring framework with balanced scoring, docking, ranking and screening powers. *Chemical Science* **2023**, *14* (30), 8129-8146.
(15) Huey, R.; Morris, G. M.; Olson, A. J.; Goodsell, D. S. A semiempirical free energy force field with charge-based desolvation. *Journal of computational chemistry* **2007**, *28* (6), 1145-1152.
(16) Quiroga, R.; Villarreal, M. A. Vinardo: A scoring function based on autodock vina improves scoring, docking, and virtual screening. *PloS one* **2016**, *11* (5), e0155183.
(17) Wang, R.; Lai, L.; Wang, S. Further development and validation of empirical scoring functions for structure-based binding affinity prediction. *Journal of computer-aided molecular design* **2002**, *16*, 11-26.
(18) Stepniewska-Dziubinska, M. M.; Zielenkiewicz, P.; Siedlecki, P. Development and evaluation of a deep learning model for protein–ligand binding affinity prediction. *Bioinformatics* **2018**, *34* (21), 3666-3674.
(19) Friesner, R. A.; Murphy, R. B.; Repasky, M. P.; Frye, L. L.; Greenwood, J. R.; Halgren, T. A.; Sanschagrin, P. C.; Mainz, D. T. Extra Precision Glide: Docking and Scoring Incorporating a Model of Hydrophobic Enclosure for Protein−Ligand Complexes. *Journal of Medicinal Chemistry* **2006**, *49* (21), 6177-6196. DOI: 10.1021/jm051256o.
(20) Schindler, C. E. M.; Baumann, H.; Blum, A.; Böse, D.; Buchstaller, H.-P.; Burgdorf, L.; Cappel, D.; Chekler, E.; Czodrowski, P.; Dorsch, D.; et al. Large-Scale Assessment of Binding Free Energy Calculations in Active Drug Discovery Projects. *J Chem Inf Model* **2020**, *60* (11), 5457-5474. DOI: 10.1021/acs.jcim.0c00900.
(21) Yang, C.; Zhang, Y. Lin_F9: A Linear Empirical Scoring Function for Protein–Ligand Docking. *J Chem Inf Model* **2021**, *61* (9), 4630-4644. DOI: 10.1021/acs.jcim.1c00737.
